# Supplementary material for: Bacterial and host factors affecting acquisition of Streptococcus pneumoniae in a murine model
Source: bioRxiv. 2026 Jan 13:2026.01.13.699243. Preprint. [Version 1] doi: 10.64898/2026.01.13.699243 (PMC12871295; doi:10.64898/2026.01.13.699243)
Supplement: Supplement 1 [file NIHPP2026.01.13.699243v1-supplement-1.pdf]

bioRxiv preprint doi: https://doi.org/10.64898/2026.01.13.699243; this version posted January 13, 2026. The copyright holder for this preprint (which was not certified by peer review) is the author/funder, who has granted bioRxiv a license to display the preprint in perpetuity. It is made available under aCC-BY 4.0 International license.

Supplemental Figures

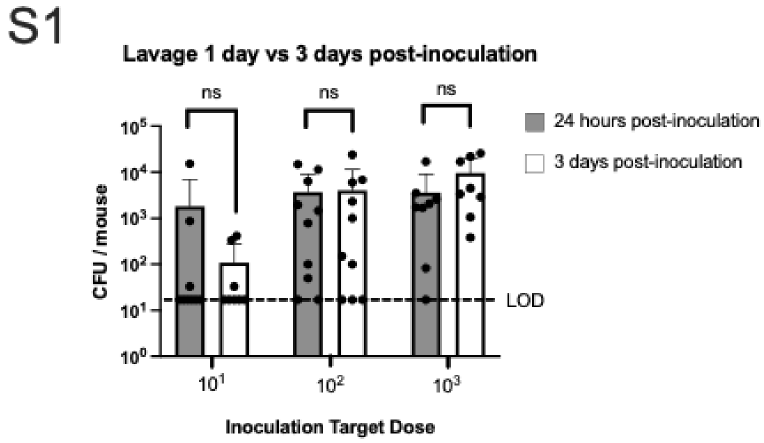

Figure S1. Effect of amount of time after challenge on rate of acquisition.

Acquisition of Spn 23F WT in lavage when mice 6-8 weeks old were euthanized and lavaged either 1 day or 3 days post-inoculation. Mice were inoculated with a dose of 10^1, 10^2, or 10^3 CFU/mouse. Statistical significance determined using Fisher's exact test comparing number of mice with at least 17 CFU/mouse to those that did not show any colonies above that limit of detection (dashed line). Each data point in the figures represents an individual animal. Horizontal bars denote the mean ± standard deviation. ns, not significant.

bioRxiv preprint doi: https://doi.org/10.64898/2026.01.13.699243; this version posted January 13, 2026. The copyright holder for this preprint (which was not certified by peer review) is the author/funder, who has granted bioRxiv a license to display the preprint in perpetuity. It is made available under aCC-BY 4.0 International license.

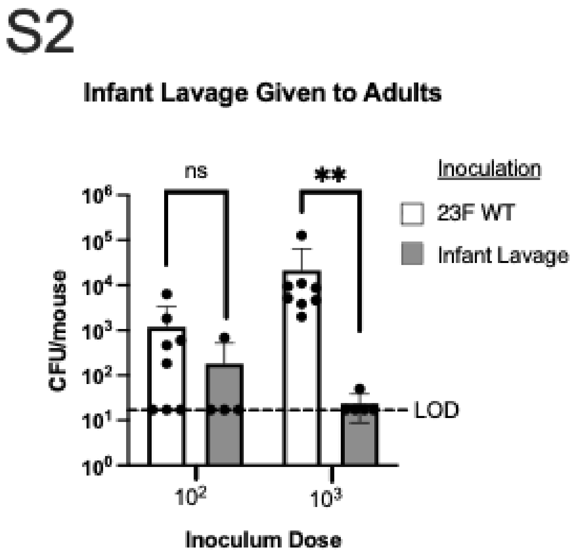

Figure S2. In vivo propagation does not improve acquisition. Infant mice 4-7 days old were inoculated with Spn 23F WT and then lavaged 3 days later. This lavage was directly inoculated into adult mice 6-8 weeks old either undiluted or at the 10^{-1} dilution, corresponding to inocula of 10^3 and 10^2 CFU/mouse, respectively. Infant lavages were plated to check correct CFU. The inoculated adults were then euthanized and lavaged 3 days post-inoculation. Results were compared to inoculation with in vitro grown 23F WT. Statistical significance determined using Fisher's exact test comparing number of mice with at least 17 CFU/mouse to those that did not show any colonies above that limit of detection (dashed line). Each data point in the figures represents an individual animal. Horizontal bars denote the mean ± standard deviation. **, p <0.01. ns, not significant.

bioRxiv preprint doi: https://doi.org/10.64898/2026.01.13.699243; this version posted January 13, 2026. The copyright holder for this preprint (which was not certified by peer review) is the author/funder, who has granted bioRxiv a license to display the preprint in perpetuity. It is made available under aCC-BY 4.0 International license.

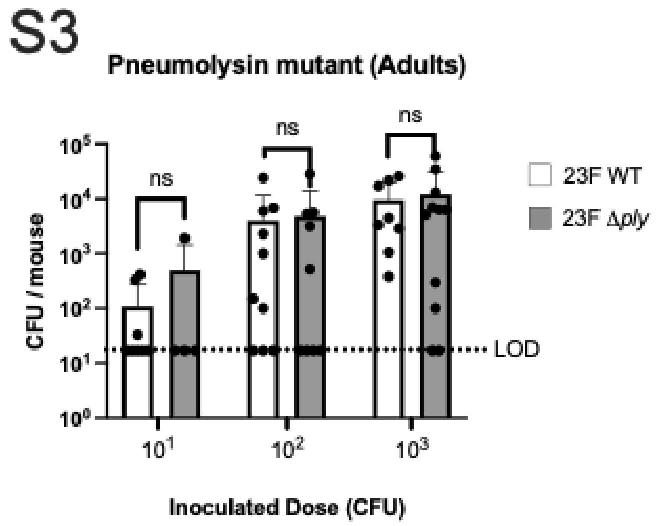

Figure S3. Knocking out pneumolysin does not affect acquisition. Acquisition of 23F WT compared to 23F strain with a pneumolysin deletion mutant (Δ*ply*) in adult mice 6-8 weeks old, lavaged 3 days after inoculation. Mice were inoculated with a dose of 10^1, 10^2, or 10^3 CFU/mouse. Statistical significance determined using Fisher's exact test comparing number of mice with at least 17 CFU/mouse to those that did not show any colonies above that limit of detection (dashed line). Each data point in the figures represents an individual animal. Horizontal bars denote the mean ± standard deviation. ns, not significant.

bioRxiv preprint doi: https://doi.org/10.64898/2026.01.13.699243; this version posted January 13, 2026. The copyright holder for this preprint (which was not certified by peer review) is the author/funder, who has granted bioRxiv a license to display the preprint in perpetuity. It is made available under aCC-BY 4.0 International license.

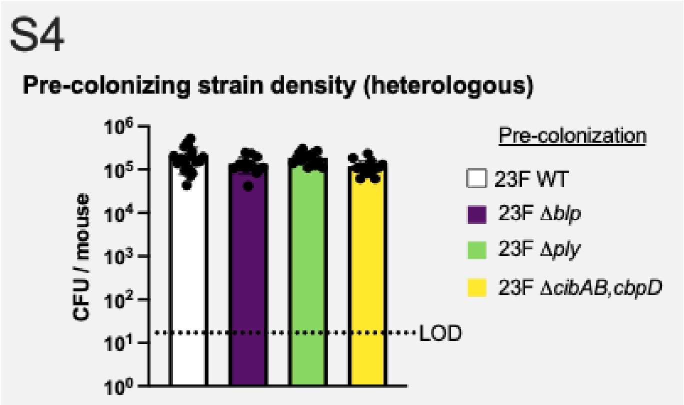

Figure S4. Equivalent colonization level for pre-colonizing strains in heterologous challenge. CFU of Spn in lavages of infant mice 3 days after challenge strain inoculation. Mice in Fig 5A. were pre-colonized with 10^3 CFU of various Spn 23F strains (wildtype (WT), *blp* locus knockout (*Δblp*), pneumolysin knockout (Δ*ply*), fratricide knockout (Δ*cibAB*,*cbpD*), and then one day later challenged with 10^1, 10^2, or 10^3 CFU doses of TIGR4. CFU of pre-colonizing strain in RT lavages shown.

bioRxiv preprint doi: https://doi.org/10.64898/2026.01.13.699243; this version posted January 13, 2026. The copyright holder for this preprint (which was not certified by peer review) is the author/funder, who has granted bioRxiv a license to display the preprint in perpetuity. It is made available under aCC-BY 4.0 International license.

S5

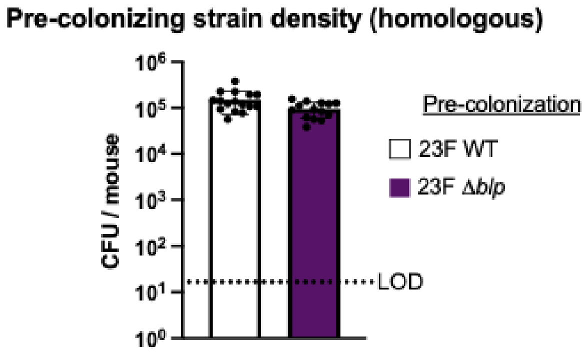

Figure S5. Equivalent colonization level for pre-colonizing strains in homologous challenge. CFU of Spn in lavages of infant mice 3 days after challenge strain inoculation. Mice in Fig 5D. were pre-colonized with 10^3 CFU of a Spn 23F strain (23F WT or 23F *blp* mutant (*Δblp*)) and one day later challenged with Spn 23F WT. 3 days post-challenge these mice were euthanized, lavaged, and the lavage was plated with 2 different antibiotics to distinguish strains. CFU of pre-colonizing strain in RT lavages shown.

bioRxiv preprint doi: https://doi.org/10.64898/2026.01.13.699243; this version posted January 13, 2026. The copyright holder for this preprint (which was not certified by peer review) is the author/funder, who has granted bioRxiv a license to display the preprint in perpetuity. It is made available under aCC-BY 4.0 International license.

S6

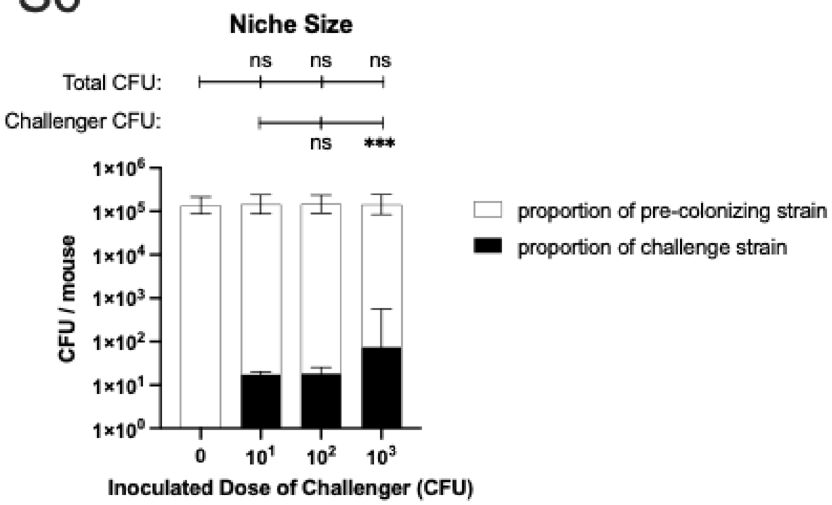

Figure S6. Competition for a limited colonization niche size. Mice in Fig, 5A were pre-colonized with various Spn 23F strains and then challenged one day later with 10^1, 10^2 or 10^3 CFU of TIGR4. The different pre-colonizing strains are grouped together as "pre-colonizing strain" and all mice are combined based on the size of the challenge strain dose. Above: Total CFU (pre-colonizing plus challenge strain) was compared to group without pre-colonization (0). CFU of the pre-colonizing strain was also compared to the group receiving the 10^1 inoculum of the challenge strain. Horizontal bars denote the mean ± standard deviation. ***, p <0.001. ns, not significant.
